# Hospitals and the Insurance of Patients

**Published:** 1911-10-21

**Authors:** 


### HOSPITALS AND THE INSURANCE OF PATIENTS.

### Some Suggestions and an Answer.

Consideration for the patient is regarded as of the first importance in every well-administered voluntary hospital, but there are risks which the managers of all Hospitals and Homes which admit patients may incur in regard to accidents, that in existing circumstances may add materially to the worries of management and the cost of maintenance. We should like to have the experience of anyone who has had to face cases like those cited in the article which follows. We will gladly render assistance to any institution in all such matters providing they send us full particulars of their requirements or experience, or both. Forewarned is forearmed, and we believe this offer of co-operation in regard to accidents and insurance cannot fail to prove welcome to some of those who habitually read The Hospital.

An insurance editor writes: A correspondent in your issue of the 7th instant asked whether hospitals and other charitable institutions for the relief of the sick are liable for accidents to patients, and whether they can insure themselves against this risk. The answer to both these questions is in the affirmative. For the benefit of readers I propose dealing with this form of risk, which, in insurance phraseology, is termed "third party insurance."

Generally speaking, the policy issued covers the insured against all liability to pay compensation for accidental injury to any person not in the insured's employ, or to their property caused on or about the insured's premises or other places agreed upon, through the fault or negligence of the insured's employees, or by any defect in the assured's ways, premises, or plant; and not only does it cover against the foregoing liability, but it covers, in addition, the insured from all legal costs incurred in defending a claim in respect of such damages, provided, of course, that such costs are incurred with the consent of the insurance office. It will thus be seen that the insurance covers not only patients but visitors, as well as the public passing by in the streets.

The following cases of claims brought against public bodies may be cited: A lady fell down the stairs of a public institution, sustained injury, and claimed £500. The claim was defeated at a cost of £20. Only quite recently a patient at one of our well-known provincial hospitals was wheeled in a bath-chair on to the parapet of the hospital. The parapet at the time was undergoing repair, and the end of it had been removed. The patient in consequence was instructed to keep still, but whether he did so or not no one will now know, for the patient was next found dead on the ground, having fallen over the parapet with the chair. This is a case that may or may not result in the hospital being mulcted in heavy damages. At any rate, if a claim was put in it would mean that the institution would have to defend it, with little or no prospect of obtaining costs, even if successful, whereas if the Hospital Committee had effected an insurance it would be fully indemnified from any liability, if any, that there may be.

The points to note in effecting this form of insurance are, first, to tabulate what risks are really insured. Generally, driving risks and those in connection with the lifts are excluded, and may be insured separately; but whether accidents in connection with the lifts are included or not, I would urge that this risk be insured separately, for the following reason: In connection with lift insurance such insurance not only includes third-party risk, but also provides for periodical inspection by experts, so that any weakness is at once detected, and thus to a great extent prevents breakdowns. Then, again, it tends to secure a more economical working cost, as any faulty working is pointed out in the reports supplied after each examination. In addition to this the policy usually provides for the making good of any breakdowns of the lift-rope's gearing and all other mechanical equipment, so that it will be seen that the cheapest course to adopt will be to insure this risk separately.

Another point to remember, and a most important one, is what limit is placed on any one accident and in any one year. Usually, the rule is to place a limit on any one accident, the total indemnity in any one year being unlimited.

Then, again, the question of law costs should be noted; in some cases the costs are included in the limit fixed, with others the law costs are over and above the limit, for it will be patent to all that if a heavy lawyer's bill has to be met, especially if it includes the costs of both sides, it will leave very little change to pay damages out of a limit of, say, £200; but if the costs are over and above the limit, then the £200 remains intact to pay the damages with. Why I put stress on this point is that certain offices fail to make any statement as to whether the costs are inclusive or not, and unless this fact is first ascertained it may result in the insured having to foot a heavy bill.

The premium charged is practically insignificant. Of course, it varies with the size of the institution and the limit desired. With regard to driving accidents, this form of insurance covers three heads, namely, damage to the public, etc., damage to the vehicle, and fatal injury to the horse. Although the third-party risk and the second may be insured alone, yet it is the best policy to effect all three. The third-party risk includes damage done to persons or their property caused by the assured's vehicles, or from bites or kicks by the horses. My remarks as to law costs apply equally to this form of insurance. The premium charged is based on the maximum number of drivers out at any one time, and varies according to the size of the town, London, of course, being rated highest.

